# Why fluctuations of conserved charges in the confining regime above $T_{ch}$ behave as if the quarks were free?

**DOI:** 10.1140/epjc/s10052-026-16479-1

**Published:** 2026-10-23

**Authors:** L. Ya. Glozman

**Affiliations:** Institute of Physics, University of Graz, 8010 Graz, Austria

## Abstract

Some cumulants of the fluctuations of conserved charges soon above the chiral crossover behave as if the quarks were free. This was taken by many as evidence of deconfinement. At the same temperatures the mesonic correlators reveal the chiral spin and *SU*(4) symmetries, indicating that the propagating degrees of freedom are massless quarks connected into color singlets by the chromoelectric confining string. These correlators are qualitatively different from the free quark gas. Here we clarify the reason for the difference. The conserved quark number densities do not propagate in time but do propagate in spatial directions. The mesonic propagators calculated in full QCD differ radically from the free quark loop (quark gas) above $T_{ch}.$ In contrast, the quark number density spatial propagator in full QCD at T≥220 MeV is very close to the free quark loop. In other words, the conserved charges do not see confinement, in contrast to the mesonic correlators. This is consistent with the well understood quark-hadron duality at T=0 in $e^+e^- \rightarrow hadrons,$ where at invariant masses above 2 GeV the cross-section in the confining regime is represented by the free quark loop plus small perturbative corrections. All these features above $T_{ch}$ but below the deconfinement temperature $T_d$ can be combined within the following microscopic picture of the stringy fluid matter. It is a medium of the overlapping strongly interacting color singlet clusters. The quark interchanges between the clusters, required by Pauli principle, make the quarks quasifree, which is reflected in fluctuations of conserved charges.

## Introduction

The fluctuations of conserved charges is one of the main topics for experimental and lattice studies in the QCD phase diagram community. Initially they were suggested as an indicator of the deconfinement at high temperatures [1–3]. For the last 20 years there have appeared a lot of lattice results on fluctuations at vanishing and small baryon chemical potentials, see e.g. [4–7] and references therein. The conserved charges do not propagate in time and consequently the strategy was chosen that bypasses the study of observables with the time evolution. Namely, the conserved charges, e.g. the baryon charge, electric charge and strangeness can be directly related to the conserved quark numbers of different flavors. The latters can be extracted from the grand canonical partition function as the first derivative with respect to the quark chemical potentials. The expectation values of the conserved quark numbers of different flavors vanish at zero chemical potentials. But their fluctuations do not vanish and can be calculated as derivatives of the conserved quark numbers with respect to the chemical potentials taken at zero. One of the results of the extensive lattice studies of the issue was that while cumulants of fluctuations are well described by the hadron gas model below the chiral restoration temperature $T_{ch} \sim 155$ MeV, some of them radically deviate from the hadron gas predictions just above $T_{ch}$ and very soon (around 220 MeV) approach the value predicted by the free quark gas. This was taken by many as an indication of deconfinement just above $T_{ch}.$

This interpretation obviously contradicts the emerged chiral spin and *SU*(4) symmetries in mesonic propagators [8–11], which point out the importance of the confining electric interaction at these temperatures (for complementary reviews on symmetries and their implications see Refs. [12, 13]). It also contradicts the results for pressure, energy density and entropy density, because a clear signal of the deconfinement and onset of the quark-gluon plasma with quasi-free quarks and gluons is the Stefan–Boltzmann $$\sim T^4$$ behavior which sets in at much larger temperatures $$ T > 400 {-} 500$$ MeV [14]. The above interpretation is also in conflict with the pion spectral function that shows very clear $$\pi ,\pi '$$ peaks above $$T_{ch}$$ [15] as well as with the bottomonium spectrum which remains the same as in vacuum at T<300 MeV (the states become broader with the temperature, however) [16].

Given symmetries and degrees of freedom the QCD phase diagram at small chemical potential was divided into three regimes (regions) connected by smooth crossovers: the hadron gas at $$T < T_{ch},$$ the stringy fluid at $$ T_{ch}< T < 3 T_{ch}$$ and the quark-gluon plasma at higher temperatures [9, 17]. The three regimes differ by the $$N_c$$ scaling of energy density, pressure and entropy density [18, 19]: $$N_c^0$$ in the hadron gas, $$N_c^1$$ in the stringy fluid and $$N_c^2$$ in the quark-gluon plasma. A very smooth crossover between the stringy fluid and the QGP is most probably centered at the deconfinement temperature $$T_d \sim 300$$ MeV of the pure glue theory [18, 20], which is supported by the deconfinement temperature in QCD extracted from the center vortices percolation [21] as well as the Hagedorn temperature $$T_H \sim 300$$ MeV within the string description [22, 23].

Recently we have published the paper [20], where we demonstrated that the fluctuations of conserved charges above $T_{ch}$ scale as $N_c^1,$ and thus are not directly sensitive to the deconfined gluons. However, the issue why the fluctuations of conserved charges behave in the confining regime above $T_{ch}$ as if the quarks were free was not addressed. In this note we explain the reason why the conserved charges do not see confinement in the confining regime. We rely on the concept of the quark-hadron duality. We do not calculate anything new, but give the missing in the literature correct interpretation of this observable. We also present an outline of the microscopic picture of the stringy fluid that is consistent with all available lattice data.

## Comparison of the QCD correlators with the free quark gas correlators

On the lattice all properties of hadrons are encoded in their temporal and spatial correlation functions. E.g. the ρ-meson is excited from the vacuum by the vector–isovector quark–antiquark bilinear $O_\Gamma (t,x,y,z)=\bar{\psi }(t,x,y,z)\gamma _\mu \frac{\boldsymbol{\tau }}{2}\psi (t,x,y,z).$

Then the Euclidean correlation functions,

1 $$\begin{aligned} C_\Gamma (t,x,y,z)=\langle O_\Gamma (t,x,y,z)\,O_\Gamma (0,{\textbf{0}})^\dagger \rangle , \end{aligned}$$

carry the full dynamical information of all isovector excitations with the ρ-meson quantum numbers. The temporal and spatial correlators in Euclidean space are defined as

2 $$\begin{aligned}  &   C_\Gamma ^t(t)=\sum _{x,y,z}C_\Gamma (t,x,y,z),\end{aligned}$$

3 $$\begin{aligned}  &   C_\Gamma ^s(z)=\sum _{x,y,t} C_\Gamma (t,x,y,z). \end{aligned}$$

The temporal correlators (2) reflect dynamics of the QCD Hamiltonian since *H* translates states in Euclidean time. The masses of hadrons are extracted from the exponential decay of the temporal correlators. The spatial correlators are connected to the dynamics of the analogous operator $H_z$ translating states in *z*-direction. At zero temperature the temporal and spatial correlators supply equivalent information, while at T>0 the information that is encoded in both correlators is different.

When we extract the ρ-meson mass from the temporal correlators we can use any μ from the set μ=1,2,3. The temporal component μ=4 is not allowed because it represents the conserved charge density that does not excite the ρ-meson and does not propagate in time. With the spatial correlators (3) only μ=1,2,4 can be used and the μ=3 component does not propagate in *z*-direction. At T=0 the temporal correlation functions with μ=1,2,3 are identical with the spatial correlation functions at μ=1,2,4, respectively.

The latter identity is violated upon increase of temperature. Still at temperatures essentially below the chiral crossover, $T \ll T_{ch},$ all three spatial correlation functions with μ=1,2,4 are equivalent. Around $T_{ch}$ a dramatic rearrangement of the spatial correlators happens. While the degenerate μ=1,2 correlators still supply the information about the ρ-meson screening masses, the conserved quark number density bilinear $\bar{\psi }(t,x,y,z)\gamma _4 \frac{\boldsymbol{\tau }}{2}\psi (t,x,y,z)$ decouples from the ρ-meson, with essentially larger slope of the correlator, and is connected to observable fluctuations of conserved charges. This is true not only for the $\bar{\psi }(t,x,y,z)\gamma _4 \frac{\boldsymbol{\tau }}{2}\psi (t,x,y,z)$ spatial correlator, but also for all other conserved quark charges.

**Fig. 1:**
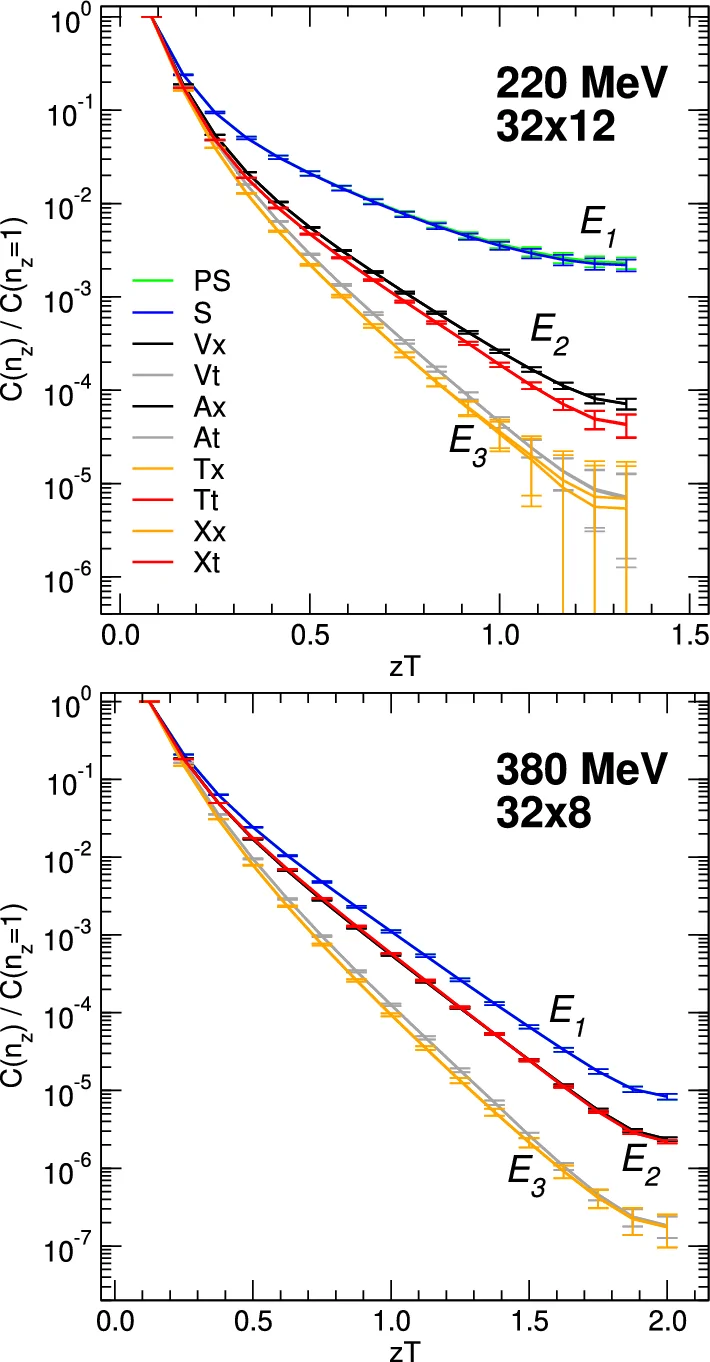
Spatial correlation functions of all possible isovector J=0,1 bilinears. From Ref. [9]

To visualize this discussion we show in Fig. 1 a complete set of spatial isovector correlators calculated in $N_F=2$ QCD at physical quark masses with chirally symmetric Dirac operator [9]. The set of local bilinear operators is presented in Table 1. We see a clear multiplet structure of the correlators at both temperatures above $T_{ch}.$ The multiplet $E_1$ consists of the scalar and pseudoscalar correlators that are connected by the $U(1)_A$ transformation. The degeneracy of both correlators indicates that the $U(1)_A$ symmetry is approximately restored and a possible remaining $U(1)_A$ breaking due to the axial anomaly is small and cannot be seen with the presented correlators. The multiplet $E_2$ represents approximate degenerate mesonic J=1 correlators that are connected by the $SU(2)_{CS}$ and *SU*(4) symmetries. The emergence of these symmetries implies that the propagating color-singlet mesons represent the chirally symmetric quarks connected by the electric string.

**Table 1:** A complete set of isovector J=0,1 operators in spatial correlators and their $U(1)_A$ and $SU(2)_L \times SU(2)_R$ transformation properties. The open vector index *k* denotes the components 1, 2, 4,  i.e. *x*, *y*, *t*

**Fig. 2:**
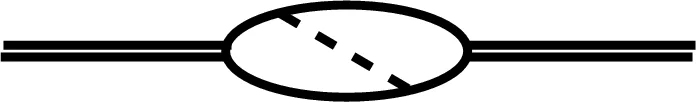
Mesonic correlator with the dashed line representing the confining electric string

The mesonic correlators from the $E_1$ and $E_2$ multiplets are schematically shown in Fig. 2, where the confining electric string between the massless quark and antiquark is depicted as dashed line and the double line is the bilinear operator $O_\Gamma$ that creates the quark–antiquark pair from the vacuum at some point.

The multiplet $E_3$ consists of four operators that include the conserved vector and axial charge density operators $V_t$ and $A_t.$ The conserved charge densities cannot propagate in time but can propagate in z-direction.

**Fig. 3:**
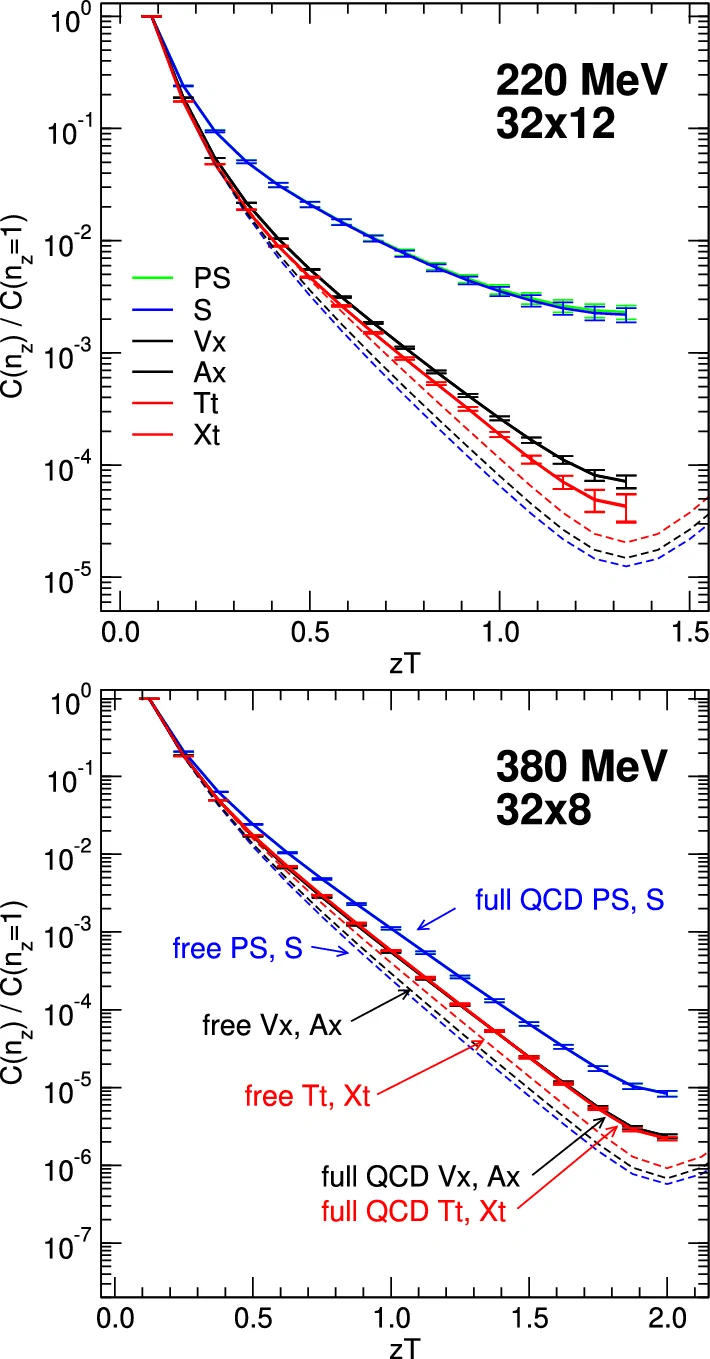
Comparison of the full QCD mesonic correlators from the multiplets $E_1$ and $E_2$ with the correlators obtained on the same lattice with free noninteracting quarks. From Ref. [9]

It is instructive to compare the mesonic correlators from the multiplets $E_1$ and $E_2$ in QCD with the correlators obtained on the same lattice with noninteracting quarks, see Fig. 3.

**Fig. 4:**
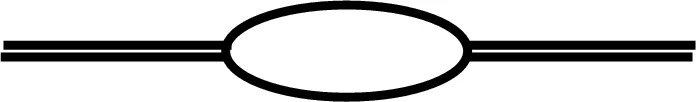
Free quark loop

The latter correlators stand for the free quark gas and consequently correspond to the quark-gluon plasma at a very high temperature where the quark-gluon interaction can be neglected. Diagramatically the free quark gas is given by the free quark loop, shown in Fig. 4.

We see a dramatic difference between the full QCD correlators and the free quark gas, especially for the J=0 mesons. The pion spectral function, extracted from the spatial correlator in Ref. [15] shows very clean $\pi , \pi '$ peaks above $T_{ch},$ which become broader with temperature. The ρ bumps with two different properties with respect to chiral symmetry, as well as the $a_1,b_1$ bumps, that could be extracted from the corresponding correlators in the $E_2$ multiplet should be expected to be essentially broader than the π peak.

**Fig. 5:**
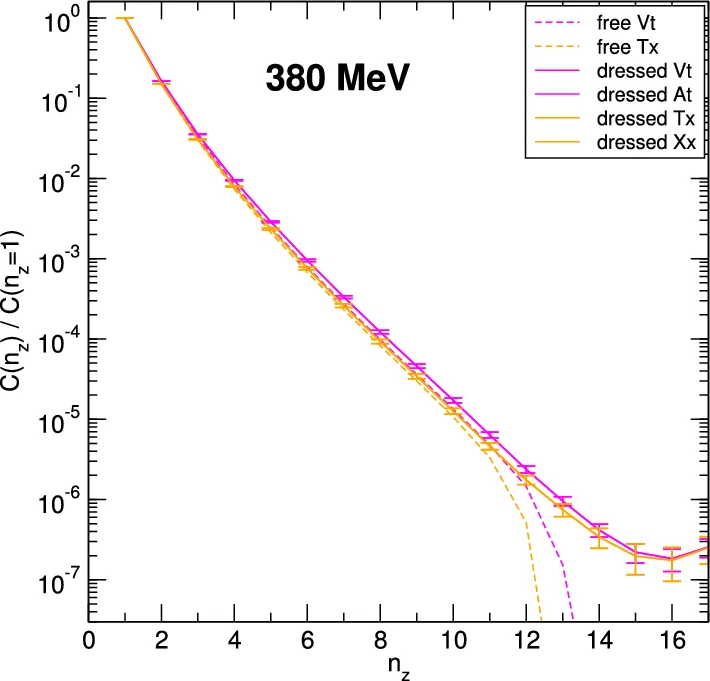
Comparison of the full QCD correlators from the multiplets $E_3$ with the correlators obtained on the same lattice with free noninteracting quarks. From Ref. [8]

Now we will compare the conserved charge density correlators in QCD at T=380 MeV with the correlators calculated with free noninteracting quarks in Fig. 5.

We observe that these correlators essentially coincide.^1^ We conclude, that contrary to the mesonic correlators, the conserved charge density, propagating in *z*-direction, is described by free quarks in the confining regime. The conserved charges do not see the confining force. This fact explains why some cumulants of the fluctuations of conserved charges behave as if the quarks were free.The conserved quark density propagators can be adequately represented by a free quark loop of Fig. 4. This effect begins at rather low temperature, soon above the chiral restoration temperature, see Fig. 6 of Ref. [11], where the full QCD correlators from the multiplet $E_3$ are compared with the free quark gas correlators at T=193 MeV. Already at such temperature the difference between the full QCD correlators $V_t$ and $A_t$ and the corresponding free quark gas correlators is insignificant. This fact explains the previous observations that some cumulants of conserved charges approach the free quark gas value already at T=220 MeV.

## Analogy to the quark-hadron duality observed at T=0 in $e^+e^- \rightarrow hadrons$

As the summary of the previous discussions we emphasize the following facts. The mesonic propagators, both spatial, discussed in the previous section, and temporal [10], demonstrate that above $T_{ch}$ the propagating degrees of freedom are the color-singlet meson-like systems, where the chirally symmetric quarks are connected into color-singlets by a confining electric string. These propagators are qualitatively different from propagators obtained with free non-interacting quarks. This observation points out the confining regime, where there are no deconfined gluons and the propagating physical degrees of freedom are only the color singlets. This is consistent with other lattice observations, discussed in the introduction. At the same time the conserved charges fluctuate very soon above $T_{ch}$ as if the quarks were free, which means that the conserved charges do not see the confining force. How could it be that in the confining regime the conserved charges are not affected by confinement?

This situation is actually not new but is well familiar and studied in detail for many years. Consider, as example, the $$e^+e^- \rightarrow hadrons$$ annihilation at T=0. The vacuum is confining, and consequently no isolated quarks and gluons can be observed. Only color singlet hadrons are registered in detectors. At the same time the inclusive cross-section above the $\rho^{\prime }$ bump, i.e., at the invariant mass of 2 GeV and above, is accurately reproduced by a free quark loop (plus some small perturbative corrections). This fact does not imply that the quarks and gluons are deconfined. It only means that the present observable is not sensitive to the confining infrared contributions [25]. This is called the quark-hadron duality. For a detailed review of the issue see Ref. [26]. At the same time the exclusive reactions, e.g. the hadron formfactors, cannot be described by perturbation theory up to very large momenta transfer, because they are always sensitive to the confining infrared contributions.

Then the analogy is rather direct. The mesonic color-singlet propagators above $T_{ch}$ but below the deconfinement temperature $T_d$ are directly sensitive to the confining force. At the same time fluctuations of conserved charges are not sensitive to the confining force and behave as if the quarks were free. A possible microscopic picture of the stringy fluid matter that is consistent with these properties is outlined in the next section.

## Microscopic picture of the stringy fluid

The first question that arises is how chiral symmetry could be restored in the confining regime? This question was answered within the microscopic calculation in the framework of the large $N_c$ manifestly confining and chirally symmetric model in 3+1 dimensions [27–34] that is similar to large $N_c$ QCD in 1+1 dimensions (’t Hooft model) [35]. It was demonstrated in Refs. [36, 37] that the chiral symmetry restoration in the confining regime happens because of Pauli blocking of the quark levels, required for the existence of the chiral condensate, by the thermal excitations of quarks and antiquarks. It was also obtained that the same Pauli blocking leads to the swelling of mesons above $T_{ch}$ several times as compared their size in vacuum (in the chiral limit the “mesons” become infinitely large). This means that the matter is a densely packed highly collective system of the overlapping color singlet “mesons” with a very small mean free path of the color singlet constituents.

While this model is only some approximation to real QCD, this qualitative microscopic picture suggests a coherent view of the stringy fluid matter that is consistent with all existing lattice data above $T_{ch}$ but below the deconfinement very smooth transition likely centered around $T_d \sim 300$ MeV:

- It is a densely packed system of the overlapping color-singlet objects.
- There are no deconfined gluons.
- It is a highly collective medium with a very small mean-free path of the color-singlet constituents.
- The propagating in time degrees of freedom are only color-singlets, in which the massless quarks and antiquarks are connected by the chromoelectric string.

Then, how could the fluctuations of conserved charges, where quarks behave as if they were free, be accommodated into this picture?

It is actually easy. Since the color singlet clusters strongly overlap, Pauli principle requires the quark interchanges between the color singlet clusters, that should be crucially important. These quark interchanges make the quarks quasi-free. Notice that it is not a free quark propagation with time. This picture should be considered as the Minkowski dual realization of the free quark loop z-propagation of conserved charge density in Euclidean space.

## Conclusions

In this paper we analysed the existing lattice data and argued that the available information above $T_{ch}$ from the correlators, equation of state, fluctuations of conserved charges, etc. is consistent with the existence of the confining regime with restored chiral symmetry at temperatures $T_{ch}< T < T_d.$ In particular, the fluctuations of conserved charges that look like the matter was a gas of free quarks, is because this observable is not sensitive to the infrared confining contributions, in contrast to the mesonic correlators. This is consistent with the well-known quark-hadron duality. The stringy fluid matter can be viewed as a system of the overlapping color-singlet clusters, with no deconfined gluons, and where the quark interchanges between the clusters, required by Pauli principle, make the quarks quasi-free. However, it is not a free propagation with time of the colored quarks. Only the color singlets, where the quarks are connected by the confining electric string, can propagate in time.

The suggested microscopic picture of the stringy fluid should be verified (falsified) in lattice simulations. A very important aspect of this picture is a huge swelling, by several times, of the pion-like excitations above the chiral crossover, as compared to the hadron gas. The size of these objects can be studied by standard lattice simulations. Another crucial aspect of the stringy fluid is the absence of deconfined gluons. This issue can be addressed on the lattice via simulations of the thermodynamical quantities (pressure, energy density and entropy density) at Nc = 5, 7 in precisely the same manner as it was done for Nc = 3. The appearance of the deconfined gluons above Td must be accompanied by the $Nc^2$ scaling, while in the stringy fluid the $Nc^1$ scaling should be expected.

## Data Availability

This manuscript has no associated data. [Authors’ comment: Data sharing not applicable to this article as no datasets were generated during the current study.]

## Footnotes

1 The diffractive behavior of the free quark correlator in Fig. 5 at $n_z > 12$ is unphysical lattice artifact related to the finite lattice volume with periodic boundary conditions in spatial directions [24]. It can be removed by combining periodic and antiperiodic boundary conditions, see Ref. [11]. Then the full QCD correlator will coincide with the free quark loop correlator at all $n_z.$

## Code Availability Statement

This manuscript has no associated code/software. [Authors’ comment: Code/Software sharing not applicable to this article as no code/software was generated during the current study.]

## References

[1] M. Asakawa, U.W. Heinz, B. Muller, Phys. Rev. Lett. **85**, 2072–2075 (2000). 10.1103/PhysRevLett.85.2072. arXiv:hep-ph/0003169 10970465

[2] S. Jeon, V. Koch, Phys. Rev. Lett. **85**, 2076–2079 (2000). 10.1103/PhysRevLett.85.2076. arXiv:hep-ph/0003168 10970466

[3] S. Ejiri, F. Karsch, K. Redlich, Phys. Lett. B **633**, 275–282 (2006). 10.1016/j.physletb.2005.11.083. arXiv:hep-ph/0509051

[4] R. Bellwied, S. Borsanyi, Z. Fodor, S.D. Katz, A. Pasztor, C. Ratti, K.K. Szabo, Phys. Rev. D **92**(11), 114505 (2015). arXiv:1507.04627 [hep-lat]

[5] S. Borsanyi, Z. Fodor, J.N. Guenther, S.K. Katz, K.K. Szabo, A. Pasztor, I. Portillo, C. Ratti, JHEP **10**, 205 (2018). 10.1007/JHEP10(2018)205. arXiv:1805.04445 [hep-lat]

[6] A. Bazavov, H.T. Ding, P. Hegde, O. Kaczmarek, F. Karsch, E. Laermann, Y. Maezawa, S. Mukherjee, H. Ohno, P. Petreczky et al., Phys. Rev. D **95**(5), 054504 (2017). 10.1103/PhysRevD.95.054504. arXiv:1701.04325 [hep-lat]

[7] J. Goswami, arXiv:2604.22514 [hep-lat]

[8] C. Rohrhofer, Y. Aoki, G. Cossu, H. Fukaya, L.Y. Glozman, S. Hashimoto, C.B. Lang, S. Prelovsek, Phys. Rev. D **96**(9), 094501 (2017). arXiv:1707.01881 [hep-lat]. [Erratum: Phys. Rev. D **99**(3), 039901 (2019)]

[9] C. Rohrhofer, Y. Aoki, G. Cossu, H. Fukaya, C. Gattringer, L.Y. Glozman, S. Hashimoto, C.B. Lang, S. Prelovsek, Phys. Rev. D **100**(1), 014502 (2019). arXiv:1902.03191 [hep-lat]

[10] C. Rohrhofer, Y. Aoki, L.Y. Glozman, S. Hashimoto, Phys. Lett. B **802**, 135245 (2020). arXiv:1909.00927 [hep-lat]

[11] T.W. Chiu, Phys. Rev. D **107**(11), 114501 (2023). arXiv:2302.06073 [hep-lat]

[12] L.Y. Glozman, Prog. Part. Nucl. Phys. **131**, 104049 (2023). arXiv:2209.10235 [hep-lat]

[13] L.Y. Glozman, arXiv:2510.14084 [hep-ph]

[14] A. Bazavov, P. Petreczky, J.H. Weber, Phys. Rev. D **97**(1), 014510 (2018). 10.1103/PhysRevD.97.014510. arXiv:1710.05024 [hep-lat]

[15] P. Lowdon, O. Philipsen, JHEP **10**, 161 (2022). arXiv:2207.14718 [hep-lat]

[16] H.T. Ding, W.P. Huang, R. Larsen, S. Meinel, S. Mukherjee, P. Petreczky, Z. Tang, JHEP **05**, 149 (2025). arXiv:2501.11257 [hep-lat]

[17] L.Y. Glozman, O. Philipsen, R.D. Pisarski, Eur. Phys. J. A **58**(12), 247 (2022). arXiv:2204.05083 [hep-ph]

[18] T.D. Cohen, L.Y. Glozman, Eur. Phys. J. A **60**(9), 171 (2024). arXiv:2311.07333 [hep-ph]

[19] L.Y. Glozman, Eur. Phys. J. C **85**(11), 1358 (2025). arXiv:2508.05277 [hep-ph] 10.1140/epjc/s10052-025-15080-2 PMC12647220 41312532

[20] T.D. Cohen, L.Y. Glozman, Eur. Phys. J. A **60**(8), 170 (2024). arXiv:2401.04194 [hep-ph]

[21] J.A. Mickley, C. Allton, R. Bignell, D.B. Leinweber, Phys. Rev. D **111**(3), 034508 (2025). arXiv:2411.19446 [hep-lat]

[22] Y. Fujimoto, K. Fukushima, Y. Hidaka, L. McLerran, Phys. Rev. D **112**(7), 074006 (2025). arXiv:2506.00237 [hep-ph]

[23] M. Marczenko, G. Kovács, L. McLerran, K. Redlich, Phys. Rev. D **112**(9), 096010 (2025). arXiv:2508.11626 [hep-ph]

[24] L.Y. Glozman, C.B. Lang, Eur. Phys. J. A **57**(6), 182 (2021). 10.1140/epja/s10050-021-00494-9. arXiv:2007.10942 [hep-lat]

[25] E.C. Poggio, H.R. Quinn, S. Weinberg, Phys. Rev. D **13**, 1958 (1976). 10.1103/PhysRevD.13.1958

[26] M.A. Shifman, Published in: *At the Frontier of Particle Physics. Handbook of QCD (Chapter 24)*, vol. 3 (World Scientific, 2001). 10.1142/9789812810458_0032. arXiv:hep-ph/0009131

[27] A.L. Yaouanc et al., Phys. Rev. D **29**, 1233 (1984)

[28] A.L. Yaouanc et al., Phys. Rev. D **31**, 137 (1985)

[29] S.L. Adler, A.C. Davis, Nucl. Phys. B **244**, 469 (1984)

[30] A. Kocic, Phys. Rev. D **33**, 1785 (1986) 10.1103/physrevd.33.1785 9956829

[31] P. Bicudo, J. Ribeiro, Phys. Rev. D **42**, 1611 (1990) 10.1103/physrevd.42.1611 10013004

[32] P. Bicudo, J. Ribeiro, Phys. Rev. D **42**, 1625 (1990) 10.1103/physrevd.42.1625 10013005

[33] F.J. Llanes-Estrada, S.R. Cotanch, Phys. Rev. Lett. **84**, 1102 (2000) 10.1103/PhysRevLett.84.1102 11017454

[34] R.F. Wagenbrunn, L.Y. Glozman, Phys. Rev. D **75**, 036007 (2007)

[35] G. ’t Hooft, Nucl. Phys. B **75**, 461 (1974)

[36] L.Y. Glozman, A.V. Nefediev, R. Wagenbrunn, Phys. Lett. B **854**, 138707 (2024)

[37] L.Y. Glozman, A.V. Nefediev, R.F. Wagenbrunn, Eur. Phys. J. C **85**(4), 462 (2025). arXiv:2410.13297 [hep-ph]

